# Supplementary figures and images for: Antibacterial activities of Miang extracts against selected pathogens and the potential of the tannin-free extracts in the growth inhibition of Streptococcus mutans
Source: PLoS One. 2024 May 8;19(5):e0302717. doi: 10.1371/journal.pone.0302717 (PMC11078415; doi:10.1371/journal.pone.0302717)

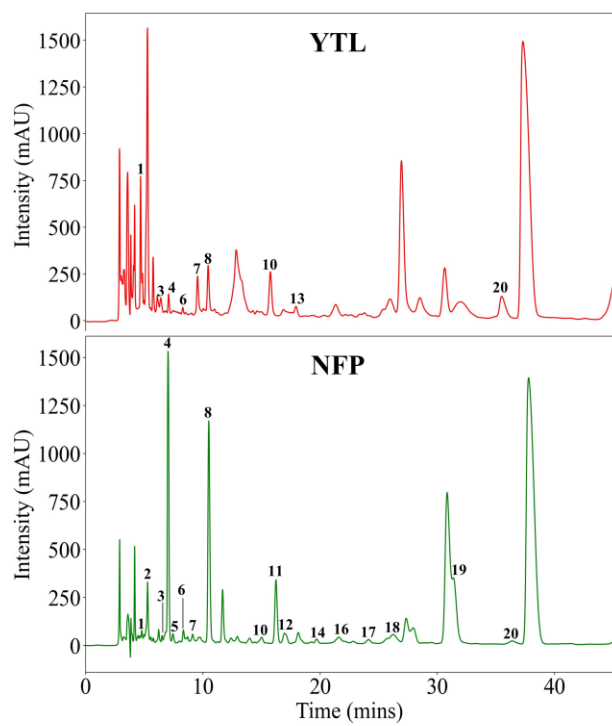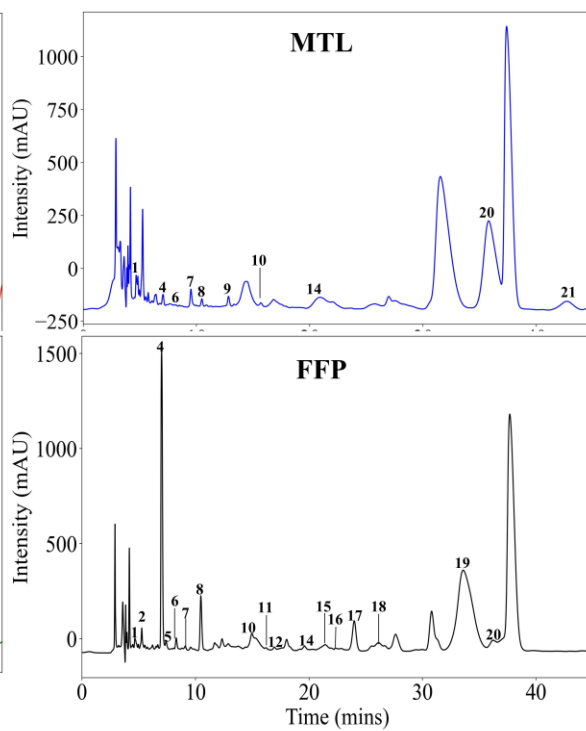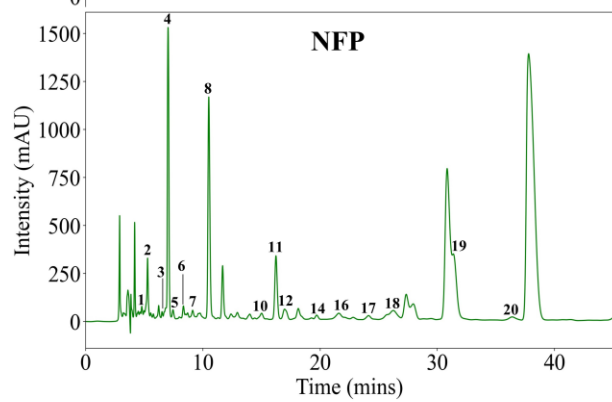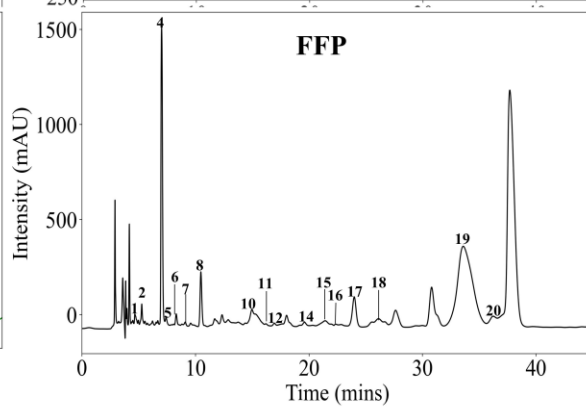

Supplement: S1 Fig — YTL: Young tea leaves, MTL: Mature tea leaves, NFP: Non-filamentous fungi growth based process fermented Miang, FFP: Filamentous fungi-growth based process fermented Miang. (PDF) [file pone.0302717.s001.pdf]
