# Supplementary material for: Antibacterial activities of Miang extracts against selected pathogens and the potential of the tannin-free extracts in the growth inhibition of Streptococcus mutans
Source: PLoS One. 2024 May 8;19(5):e0302717. doi: 10.1371/journal.pone.0302717 (PMC11078415; doi:10.1371/journal.pone.0302717)
Supplement: S1 Table — aAbbreviations are listed as follows: OA; organic acid; F3, flavan-3-ol; FL, flavonol/flavone; PA, phenolic acid; PP, Polyphenol; AA, amino acid. bRT: Retention time All acquisitions were carried out in the positive mode (m/z, [M + H] +) using LC-MS (at 210 and 270 nm wavelengths). (PDF) [file pone.0302717.s002.pdf]

Peak	Class[a]	RT[b] (mins)	MW	Theoretical m/z	$[M + H]^+$ m/z	Mass error (mDa)	Formula	Tentative identification	Sample
1	PA	4.70	354.31	355.1024	354.0951	0.069	$C_{16}H_{18}O_9$	Chlorogenic acid	YTL, MTL, NFP, FFP
2	PA	5.27	368.34	369.1180	368.1107	0.019	$C_{17}H_{20}O_9$	5-Feruloylquinic acid	NFP, FFP
3	FL	6.46	302.24	303.0499	302.0427	-0.074	$C_{15}H_{10}O_7$	Quercetin	YTL, NFP
4	FL	7.04	480.38	481.0977	480.0904	-0.007	$C_{21}H_{20}O_{13}$	Myricetin-3-galactoside	YTL, MTL, NFP, FFP
5	PA	7.50	338.31	339.1074	338.1002	0.084	$C_{16}H_{18}O_8$	3-p-Coumaroylquinic acid	NFP, FFP
6	AA	8.27	129.11	130.0499	129.0426	-	$C_5H_7NO_3$	Pyroglutamic acid	YTL, MTL, NFP, FFP
7	FL	9.06	626.52	627.1556	626.1483	0.000	$C_{27}H_{30}O_{17}$	Quercetin dihexoside	YTL, MTL, NFP, FFP
8	PA	10.17	516.45	517.1341	516.1268	0.003	$C_{25}H_{24}O_{12}$	Dicaffeoylquinic acid 3	YTL, MTL, NFP, FFP
9	F3	13.32	458.37	459.0922	458.0849	0.029	$C_{22}H_{18}O_{11}$	Epigallocatechin gallate	MTL
10	PA	15.16	516.45	517.1341	516.1268	0.035	$C_{25}H_{24}O_{12}$	Dicaffeoylquinic acid 1	YTL, MTL, NFP, FFP
11	OA	16.59	90.08	91.0390	90.0317	-	$C_3H_6O_3$	Lactic acid	NFP, FFP
12	PA	17.30	192.17	193.0707	192.0634	0.325	$C_7H_{12}O_6$	Quinic acid	NFP, FFP
13	F3	17.94	290.27	291.0863	290.0790	0.023	$C_{15}H_{14}O_6$	Epicatechin	YTL
14	PA	20.59	258.19	259.0561	258.0488	-0.003	$C_9H_{10}N_2O_7$	Uronic acid	MTL, NFP, FFP
15	FL	22.33	726.63	727.2080	726.2007	-0.018	$C_{32}H_{27}O_{14}$	Apigenin-6-*C*-glucosyl-8-*C*-arabinoside	FFP
16	PA	22.73	170.12	171.0288	170.0215	-0.144	$C_7H_6O_5$	Gallic acid	NFP, FFP
17	PA	24.06	302.19	303.0135	302.0063	0.074	$C_{14}H_6O_8$	Ellagic acid	NFP, FFP
18	PP	26.57	126.11	127.0390	126.0317	0.070	$C_6H_6O_3$	Pyrogallol	NFP, FFP
19	PA	35.81	88.11	89.0597	88.0524	-	$C_4H_8O_2$	Butyric acid	NFP, FFP
20	FL	37.54	464.38	465.1026	464.0955	0.003	$C_{21}H_{20}O_{12}$	Quercetin-3-glucoside	YTL, MTL, FFP
21	F3	42.81	306.27	307.0812	306.0740	0.109	$C_{15}H_{14}O_7$	Gallocatechin	MTL
