# Supplementary material for: Antibacterial activities of Miang extracts against selected pathogens and the potential of the tannin-free extracts in the growth inhibition of Streptococcus mutans
Source: PLoS One. 2024 May 8;19(5):e0302717. doi: 10.1371/journal.pone.0302717 (PMC11078415; doi:10.1371/journal.pone.0302717)
Supplement: Attachment — Submitted filename: Comments PLos one.pdf [file pone.0302717.s004.pdf]

Comments

	Comments
Title	This title suitable with the article content
Abstract	Acceptable. However, suggest to include problem statement
Introduction	Line 58- The statement mention that resilient efforts to combat infectious diseases and pathogenic bacteria (such as Bacillus 58 cereus and Escherichia coli) that cause foodborne illnesses and/or pose serious problems to 59 human health is a current research quest. However, the reference use to support the statement that mention 'current research' is on 2017. Pls find latest references.<br><br>-The introduction focus on foodborne pathogens issue. However this research do analysis on Streptoccus mutants. Does this strain is classify as foodborne pathogens?<br><br>-State the justification on the selected pathogens
Methodology	Line 112- Justification on selection of acetone as extraction solvent. We have several types of solvent that normally used in extraction including methanol, ethanol, water and so on.<br><br>-There is no procedures on the adjustment of extract pH
result	Line 245 and 251. The figures title are similar. Revise the title by adding the pH use cause the both figure display antibacterial activity of extract at different pH
Discussion	Discussion was clearly discuss with appropriate support statement based on previous studies
Conclusion	Conclusion is reflect the finding and the article title
