# Supplementary material for: Antibacterial activities of Miang extracts against selected pathogens and the potential of the tannin-free extracts in the growth inhibition of Streptococcus mutans
Source: PLoS One. 2024 May 8;19(5):e0302717. doi: 10.1371/journal.pone.0302717 (PMC11078415; doi:10.1371/journal.pone.0302717)
Supplement: Attachment — Submitted filename: PONE-D-23-35234_Comments.docx [file pone.0302717.s003.docx]

The manuscript PONE-D-23-35234 entitled “Antibacterial activities of Miang extracts against selected pathogens and the potential of the tannin-free extracts in the growth inhibition of Streptococcus mutans” has described solvent extraction of Miang (fermented tea leaves) and tea leaves, raw material for Miang production and determination of antibacterial activities of the obtained extracts, and those treated with tannin-free against selected bacterial pathogens, examination of compounds. The results have found that tannin-free Miang extracts showed more potential to inhibit growth of pathogens, specifically *Streptococcus mutans* than tannin-free tea leaf extracts. In addition, the manuscript has described analysis of the extracts by HPLC-MS in order to examine the chemical compounds that are relevant to growth inhibition of *Streptococcus mutans*. The identified compounds were used to determine interactions between each identified compound and sortase A enzyme, the key enzyme associated with biofilm formation of *S. mutans*. The authors conclude that the tannin-free Miang extracts could be used as a natural active pharmaceutical ingredient for applications in oral hygiene products.

After the evaluation, this manuscript presents sufficient experimental designs and statistical analysis. However, there is unclear information and errors. To meet the standard manuscript for publication, the current form of the manuscript must be substantially revised. The following comments and suggestions would help increase the completement of the manuscript.

General comments and suggestions

Please check the writing format of the scientific name of all microorganisms. There are “*Streptococcus*” and “*Salmonella*” which can be abbreviated as “*S*.”. This would make readers confused. It is suggested to present “*S*.” for *Streptococcus* mutans and “*Sm*”. for *Salmonella*.

To describe *Salmonella enterica* serovar Typhimurium, it is recommended to state it as “*Salmonella* var. Typhimurium” or “*Salmonella* Typhimurium” or “*Sm.* Typhimurium”. Please correct it in the whole manuscript.

Please indicate strain to all microorganisms used in this study.

Full name of a microorganism must be present for the first time of the main section and then it should be abbreviated. However, when the authors state it as a subject of the sentence, it is recommended to state its full name.

It is confusing when the authors state NFP and FFP as Miang because either NFP or FFP is a process of Miang fermentation. It is recommended to state it as “NFP Miang” or “FFP Miang”. Please also correct these words throughout the manuscript.

Comma symbol (,) must be added after a specific word such as respectively, especially, etc.,

Considering the type of extracts used in this study and their consistency, please reconsider these words: non-neutral, neutral, acidic, and pH 7. If tea leaf extracts (YTL and MTL) and NFP and FFP Miang extracts are generally acidic, it could state them as “non-neutralized extracts”. After the authors have neutralized the acidic extracts, it could state them as “neutralized extracts”.

Due to Miang is made from tea leaves, it is relevant to express only young tea leaves or mature tea leaves rather than unfermented young tea leaves and unfermented mature tea leaves, respectively (Line 228 and others in discussion section).

Please italicize p of p-value and t of t-value in whole manuscript.

It is more relevant to replace “PVPP-treated extract’ supernatant” by “PVPP-treated extract”. (lines 148, 398, 408, …)

Major and minor comments

Introduction section, add more information about role of sortase A enzyme in dental plaque formation. This information would at least let readers primarily understand why the authors chose molecular docking to investigate interactions between active compounds in Miang extracts and the sortase A enzyme.

Line 109-107 how did the authors express the unit of all extract? Is it mg/mL total polyphenol content or mg/mL total tannin content, or mg extract/mL, etc., Please add more information.

Line 127, is it non-neutralized Miang extracts?

Line 139, is it neutralized Miang extracts? Please also consider as above mentioned.

Line 133, indicate diameter of borer.

Line 134, indicate temperature.

Line 163, remove “be”

Line 172, edit to “*Streptococcus mutans*” when it was first used as the subject.

Line 173, edit to “an optical density at 600 nm (OD_600_)”

Liness 223, 247, and 253 add the strain of *S. mutans*.

Lines 230 and 239 how did the authors calculate percentages of inhibition? Please clarify.

Table 1, the authors can categorize the extracts into two groups including non-neutralized (acidic) and neutralized extracts. Each group consists of YTL, MTL, NFP, and FFP.

Line 236-237, different alphabets represent significant differences vs control. The control was just water. If the authors compared the experimental values to the control, extract samples exhibited the clear zone will have significant differences. From the point of view, multiple comparisons were made after ANOVA results showed significant differences (*p* < 0.05). It is supposed that all pairwise comparisons were used to analyze the data within the same row, not just compared with the control. It is noted that data with positive results are always significant when they are compared with the control. Please consider this comment for Fig 3., Table 2, Fig 5, and Fig 6.

Lines 245 and 251, it is recommended to revise the figure legend as follows:

Fig 1. Antibacterial activities of non-neutralized tea leaf extracts and non-neutralized Miang extracts against *Bacillus cereus* TISTR 747 (A), *Escherichia coli* ATCC 22595 (B), *Salmonella* Typhimurium TISTR 292 (C), *Streptococcus mutans* DMST 18777 (D). Sterile water was used as the control.

Fig 2. Antibacterial activities of neutralized tea leaf extracts and neutralized Miang extracts against *Bacillus cereus* TISTR 747 (A), *Escherichia coli* ATCC 22595 (B), *Salmonella* Typhimurium TISTR 292 (C), *Streptococcus mutans* DMST 18777 (D). Sterile water was used as the control.

Lines 256-277, it is recommended to initially describe line 257-260 (Antibacterial quantitative assays showed …….at acidic and neutral extracts’ pH respectively). However, the phrase “at acidic and neutral extracts’ pH respectively” should be revised to proper format as previously suggested. For example, Antibacterial quantitative assays showed an overall better MIC and MBC for NFP and FFP Miang extracts compared to the YTL and MTL when they were tested against all the pathogens, respectively.

Line 278, it is recommended to revise the figure legend as follows:

Fig 3. Antibacterial activities of non-neutralized and neutralized extracts of Miang and tea leaves against *Bacillus cereus* TISTR 747 (A), *Escherichia coli* ATCC 22595 (B), *Salmonella* Typhimurium TISTR 292 (C), *Streptococcus mutans* DMST 18777 (D).

Line 282, Different asterisk (*) represent significant differences vs control. What was the control of this experiment? Is it the initial cell concentration or just sterile water? This phrase could be modified to “Different asterisk symbols * and ** represent significant differences of MIC and MBC, respectively (*p* < 0.05)”.

Line 289, please consider modifying description of table 2 as follows:

Table 2. The inhibition zone of tannin-free extracts of Miang and tea leaves against *Streptococcus mutans* DMST 18777. Avoid extension of figure description.

Line 293 the comment is similar to that is given for Line 236-237

Line 296, it is unnecessary to add Fig 4 because the authors have already displayed the results in Table 2.

Lines 309-314 it is necessary to compare effect of tannin-free extracts and their original extract (in the presence of tannin)

Lines 384-386 *Streptococcus mutans* has been reported to recruit sucrose-dependent mechanisms where glucosyltransferase (GTF) reacts with sucrose and convert into dextran polymer or glucan that can be further bound with glucan-binding proteins (GBP) which leads to the formation of dental plaque. Please consider modifying the sentence.

Lines 411-425 please shorten principal discussion about role of sortase A in plaque formation but discuss more about finding of this study. How could active compounds from PVPP-treated Miang extracts inhibit sortase A? Are there any literatures reported active compounds that have potential to inhibit the enzyme?

Line 438-440 the sentence in the conclusion is not relevant to what the author proposes in introduction section (Lines 51-53, and 90-93).
