# Supplementary material for: Antibacterial activities of Miang extracts against selected pathogens and the potential of the tannin-free extracts in the growth inhibition of Streptococcus mutans
Source: PLoS One. 2024 May 8;19(5):e0302717. doi: 10.1371/journal.pone.0302717 (PMC11078415; doi:10.1371/journal.pone.0302717)
Supplement: Attachment — Submitted filename: Response to Reviewers.doc [file pone.0302717.s005.doc]

**POINT BY POINT RESPONSE TO THE EDITOR AND REVIEWERS’ COMMENTS ON THE MANUSCRIPT (PONE-D-23-35234).**

Thank you for giving us the opportunity to revise the manuscript and the willingness to accept the article after the major revision. We have considered each of the comments given by both editor and reviewers and have provided revisions/responses to each comment in a point-by-point pattern. Please note that all corrections by the authors are made using Tracked changes and saved as “Revised document with Track changes”. Line numbers (LNs) are also included in the point by point responses to reviewers for efficient tracking of corrections.

**Editor**

**General Response:** We are grateful to the editor for the kind comments and suggestions meant to improve the manuscript.

**Comment 1:**  Please include a rebuttal letter that responds to each point raised by the academic editor and reviewer(s). You should upload this letter as a separate file labelled 'Response to Reviewers'.

**Response 1:** A rebuttal letter labelled 'Response to Reviewers' has been included per directive of the editor

**Comment 2:**  Please include a marked-up copy of your manuscript that highlights changes made to the original version. You should upload this as a separate file labelled 'Revised Manuscript with Track Changes'.

**Response 2: “**Track changes” in MS word has been used to highlight the revisions and the file has been saved as 'Revised Manuscript with Track Changes'.

**Comment 3:**  Please an unmarked version of your revised paper without tracked changes. You should upload this as a separate file labelled 'Manuscript'.

**Response 3:** A clean copy of the Manuscript devoid of highlights and tracked changes has been labelled and saved as 'Manuscript'.

**Reviewer 1**

**General Response:** We are grateful to the reviewer for the kind comments and efforts to make the manuscript better. All the corrections have been implemented and can be confirmed easily in the file saved as “Revised manuscript with track changes”. Line numbers (LNs) are also included in the point by point responses to reviewers for efficient tracking of corrections.

**Comment 1:** Please check the writing format of the scientific name of all microorganisms. There are “Streptococcus” and “Salmonella” which can be abbreviated as “S.”. This would make readers confused. It is suggested to present “S.” for Streptococcus mutans and “Sm”. for Salmonella. To describe Salmonella enterica serovar Typhimurium, it is recommended to state it as “Salmonella var. Typhimurium” or “Salmonella Typhimurium” or “Sm. Typhimurium”. Please correct it in the whole manuscript. Please indicate strain to all microorganisms used in this study. Full name of a microorganism must be present for the first time of the main section and then it should be abbreviated. However, when the authors state it as a subject of the sentence, it is recommended to state its full name.

**Response 1:** Thank you for the comment. The writing format for the scientific names have been modified as suggested and the corrections was applied throughout the manuscript.

**Comment 2:** It is confusing when the authors state NFP and FFP as Miang because either NFP or FFP is a process of Miang fermentation. It is recommended to state it as “NFP Miang” or “FFP Miang”. Please also correct these words throughout the manuscript.

**Response 2: “**NFP” and “FFP” has been clarified and changed throughout the manuscript to “NFP Miang” or “FFP Miang” (**LNs 43, 47, 52, 60, 98, 99, 107, 109, 114, 233, 234, 236, 249, 270,273, 277, 283, 287, 291-293, 342, 352, 371, 376, 380, 392, 396, 412, 435-436, 441-442 and 471**) as recommended.

**Comment 3:** Comma symbol (,) must be added after a specific word such as respectively, especially, (**LNs 70, 152, 161, 286, 292, 303, 381, 413 and 480**) etc.

**Response 3**: Comma symbols have been added as suggested for specific words.

**Comment 4:** Considering the type of extracts used in this study and their consistency, please reconsider these words: non-neutral, neutral, acidic, and pH 7. If tea leaf extracts (YTL and MTL) and NFP and FFP Miang extracts are generally acidic, it could state them as “non-neutralized extracts”. After the authors have neutralized the acidic extracts, it could state them as “neutralized extracts”.

**Response 4**: Thank you for the comment. “Non-neutral extract” and “neutral extract” were replaced with “non-neutralized extracts” and “neutralized extracts” respectively (**LNs 131-132, 144, 253, 262, 271, 274, 284, 287, 289, 295, 322 and 477**). However, words such as non-neutral pH, and neutral pH were not altered.

**Comment 5:** Due to Miang is made from tea leaves, it is relevant to express only young tea leaves or mature tea leaves rather than unfermented young tea leaves and unfermented mature tea leaves, respectively (Line 228 and others in discussion section).

**Response 5**: The word “unfermented” in **LNs 234, 359, 373, 411 and 440**, has been erased due to its redundancy. Thank you for the suggestion.

**Comment 6:** Please italicize p of p-value and t of t-value in whole manuscript.

**Response 6**: All p-values found in **LNs 227, 244-245, 249, 278, 280, 293, 303, 308, 316, 329, 335 and 348**, have been italicized.

**Comment 7:** It is more relevant to replace “PVPP-treated extract’ supernatant” by “PVPP-treated extract”. (Lines 148, 398, 408 …).

**Response 7**: Thank you for the comment. “PVPP-treated extract’ supernatant” has been changed to “PVPP-treated extract” (**LNs 160, 180, 206, 330, 339-340, 433, 438 and 446**) throughout the manuscript.

**Comment 8:**  Introduction section, add more information about role of sortase A enzyme in dental plaque formation. This information would at least let readers primarily understand why the authors chose molecular docking to investigate interactions between active compounds in Miang extracts and the sortase A enzyme.

**Response 8:** Additional information has been included in the Introductory part as recommended: **LNs 80-82**, “The biofilm formation is mediated through sortase A enzyme which anchors surface proteins on bacterial cell walls to allow host attachment by *S. mutans* biofilms. Consequently, these events lead to the formation of dental plaque, demineralization of enamel and leaching of teeth components [14,15]”.

**Comment 9:** Line 109-107 how did the authors express the unit of all extract? Is it mg/mL total polyphenol content or mg/mL total tannin content, or mg extract/mL, etc., Please add more information.
**Response 9**: The unit of the extract was expressed as mg extract/mL as indicated now in **LN 121** as suggested.

**Comment 10:** Line 127, is it non-neutralized Miang extracts? Line 139, is it neutralized Miang extracts? Please also consider as above mentioned.

**Response 10**: Thank you for the comment, the previous recommendation (comment 4) also relates to this and has been judiciously implemented throughout the manuscript.

**Comment 11:** Line 133, indicate diameter of borer. Line 134, indicate temperature. Line 163, remove “be” Line 172, edit to “Streptococcus mutans” when it was first used as the subject. Line 173, edit to “an optical density at 600 nm (OD600)” Lines 223, 247, and 253 add the strain of S. mutans.

**Response 11:** The diameter of the borer: “6mm diameter”, has been indicated (**LN 138**). Temperature has been indicated on LN 140 “incubated for 24 h at 37 ^o^C”. “be” has been removed from **LN 168**. **Line 177**, has been edited to “*Streptococcus mutans* DMST 18777” due to its use here as the subject. **LN 178** has been edited to “to attain an optical density at 600 nm (OD600) of ~1”. The strain of *S. mutans* has been included in **LNs 235, 291 and 308.** All the corrections have been implemented and can be confirmed easily in the file saved as “Revised manuscript with track changes”.

**Comment 12:** LNs 230 and 239 how did the authors calculate percentages of inhibition? Please clarify.

**Response 12:** Now LNs 236 and 247, % Contribution of neutralized compounds (Table 1) was expressed as (Zone of inhibition for neutralized extract/ Zone of inhibition for non-neutralized extract) x 100%. Then, contribution of organic acids = 100 % ˗ % Contribution of neutralized compounds.

**Comment 13:** Table 1, the authors can categorize the extracts into two groups including non-neutralized (acidic) and neutralized extracts. Each group consists of YTL, MTL, NFP, and FFP.

**Response 13**: Thank you for the comment, the change was duly noted (**LNs 238-245**) and incorporated in the table.

**Comment 14:** Line 236-237, different alphabets represent significant differences vs control. The control was just water. If the authors compared the experimental values to the control, extract samples exhibited the clear zone will have significant differences. From the point of view, multiple comparisons were made after ANOVA results showed significant differences (p < 0.05). It is supposed that all pairwise comparisons were used to analyze the data within the same row, not just compared with the control. It is noted that data with positive results are always significant when they are compared with the control. Please consider this comment for Fig 3., Table 2, Fig 5, and Fig 6.

**Response 14:** Thank you for the comment, the comment was considered and a modification (“Different alphabets represent significant differences (*p*<0.05, Turkey’s test).”) was applied accordingly to the affected figures and tables mentioned above (**LNs 244, 302, 316-317, 335 and 347**).

**Comment 15:** Lines 245 and 251, it is recommended to revise the figure legend as follows:

Fig 1. Antibacterial activities of non-neutralized tea leaf extracts and non-neutralized Miang extracts against *Bacillus cereus* TISTR 747 (A), *Escherichia coli* ATCC 22595 (B), *Salmonella* Typhimurium TISTR 292 (C), *Streptococcus mutans* DMST 18777 (D). Sterile water was used as the control.

Fig 2. Antibacterial activities of neutralized tea leaf extracts and neutralized Miang extracts against *Bacillus cereus* TISTR 747 (A), *Escherichia coli* ATCC 22595 (B), *Salmonella* Typhimurium TISTR 292 (C), *Streptococcus mutans* DMST 18777 (D). Sterile water was used as the control.

**Response 15:** Thank you for the comment. The Figure title has been revised as recommended (**LNs 253-269**).

**Comment 16:** Lines 256-277, it is recommended to initially describe line 257-260 (Antibacterial quantitative assays showed …….at acidic and neutral extracts’ pH respectively). However, the phrase “at acidic and neutral extracts’ pH respectively” should be revised to proper format as previously suggested. For example, Antibacterial quantitative assays showed an overall better MIC and MBC for NFP and FFP Miang extracts compared to the YTL and MTL when they were tested against all the pathogens, respectively.

**Response 16:** Now LNs 272 – 275, the MIC and MBC was initially defined in Lines 151 and 152. However, it has been modified to “Antibacterial quantitative assays showed an overall better MIC (< 3 log decrease in CFU/mL) and MBC (> 3 log decrease in CFU/mL) for NFP and FFP Miang compared to the young (YTL) and mature tea leaves (MTL) against all the pathogens at non-neutralized and neutralized pH of the extracts respectively”.

**Comment 17:** Line 278, it is recommended to revise the figure legend as follows:

Fig 3. Antibacterial activities of non-neutralized and neutralized extracts of Miang and tea leaves against *Bacillus cereus* TISTR 747 (A), *Escherichia coli* ATCC 22595 (B), *Salmonella* Typhimurium TISTR 292 (C), *Streptococcus mutans* DMST 18777 (D).

**Response 17:** The figure title has been revised as recommended (LN 295).

**Comment 18:** Line 282, Different asterisk (*) represent significant differences vs control. What was the control of this experiment? Is it the initial cell concentration or just sterile water? This phrase could be modified to “Different asterisk * and ** represent significant differences of MIC and MBC, respectively (p < 0.05)”.

**Response 18:** The initial cell concentration and sterile water were both incorporated in the experimental design. However, “Different asterisk * and ** represent significant differences of MIC and MBC, respectively (*p*<0.05)” better captures the multiple comparison from Turkey’s post hoc test. Thank you very much for this comment (**LN 302**).

**Comment 19:** Line 289, please consider modifying description of table 2 as follows:

Table 2. The inhibition zone of tannin-free extracts of Miang and tea leaves against *Streptococcus mutans* DMST 18777. Avoid extension of figure description.

**Response 19:** Thank you for the comment. The simpler description has been implemented (**LN 310**) as suggested.

**Comment 20:** Line 293 the comment is similar to that is given for Line 236-237. Line 296, it is unnecessary to add Fig 4 because the authors have already displayed the results in Table 2.

**Response 20:** “Different alphabets…” indicating the multiple comparisons from Turkey’s test have been modified throughout the manuscript as recommended. However, we feel some readers resonate more with figures so we showed the fig 2 in addition to the table (which shows the statistical difference) in a bid to accelerate readers’ understanding of the result.

**Comment 21:** Lines 309-314 it is necessary to compare effect of tannin-free extracts and their original extract (in the presence of tannin)

**Response 21:** Thank you for the comment, the effect of tannin-free extracts and their original extracts were compared in table 2 where independent sample T-test was used to explain the difference. Against the *S. mutans* DMST 18777 biofilms, the point of this publication was to explore the potential of tannin free extracts in the growth inhibition of the biofilms. The justification for this was mentioned in the introduction (Lines 90 – 97).

**Comment 22:** Lines 384-386 Streptococcus mutans has been reported to recruit sucrose-dependent mechanisms where glucosyltransferase (GTF) reacts with sucrose and convert into dextran polymer or glucan that can be further bound with glucan-binding proteins (GBP) which leads to the formation of dental plaque. Please consider modifying the sentence.

**Response 22:** Lines 394 – 397), the sentence has been modified to “*Streptococcus mutans* has been reported to recruit sucrose-dependent mechanisms where glucosyltransferase (GTF) mediates a reaction with glucan-binding proteins (GBP) to cause the formation of dental plaques [49–51]. Interestingly, tannins have been reported to inhibit GTF-mediated synthesis of insoluble glucans thus suppressed *S. mutans* proliferation”.

**Comment 23:** Lines 411-425 please shorten principal discussion about role of sortase A in plaque formation but discuss more about finding of this study. How could active compounds from PVPP-treated Miang extracts inhibit sortase A? Are there any literatures reported active compounds that have potential to inhibit the enzyme?

**Response 23:** The discussion was made to be as concise as possible (Lines 448 – 465). And a Paper which was recently published (December, 2022) was cited which mentioned related active compounds that have found use in maintaining oral health. Line 466 – 469, “Medicinal plants rich in flavonoids (including kempferol, apigenin and quercetin) and tannins (such as ellagitannins and proanthocyanidins) have been shown to contribute to the maintenance of oral health and are therefore applied as additives or main ingredients in mouthwashes or toothpastes [59]”.

**Comment 24:** Line 438-440 the sentence in the conclusion is not relevant to what the author proposes in introduction section (Lines 51-53, and 90-93).

**Response 24:** The sentence has been modified to “Overall, the result showed the potential of these extracts to be enriched, optimized and possibly find future applications as a mainstay in combating bacterial pathogens and as potential ingredients in mouthwashes” (484 – 486).

**Reviewer 2**

**General Comment:** It was advantageous to discover a plant extract that inhibited pathogen growth. Several concerns, however, needed to be elucidated in order to have a better knowledge of Miang extracts' antibacterial activity.

**General Response:** Thank you very much for the comment. Your concerns will be given due attention and addressed appropriately. All corrections by the authors are made using Tracked changes and saved as “Revised document with Track changes”. Line numbers (LNs) are also included in the point by point responses to reviewers for efficient tracking of corrections.

**Comment 1:** It was unclear how the authors obtained the quantities of Miang extracts utilized in Table 1 to establish zones of inhibition against bacteria.

**Response 1**: Thank you for the comment. **LNs 114 – 122** summarizes the preparation of Miang extract. Briefly, 5g of each extract was extracted in 100 mL of 80% acetone. The obtained solution was filtered and evaporated using a rotary evaporator (N-1000 Eyela Rotary Evaporator; Tokyo, Japan) at 40 *◦*C, concentrated, and dissolved each in 20 mL deionized (DI) water (RCI Labscan Co., Ltd., Bangkok, Thailand) thus reaching a final concentration of 109.5 mg/mL (YTL), 100.5 mg/mL (MTL), 74.5 mg/mL (NFP Miang) and 91.5 mg/mL (FFP Miang).

**Comment 2:** Please double-check whether it was Turkey's or Tukey's test (line 237).

**Response 2:** Thank you for the comment. “Turkey's” have been replaced with “Tukey's” throughout the manuscript (**LNs 244, 336 and 348**).

**Comment 3:** Only triplicate samples used to run a t-test comparing tannin rich extract to tannin-free extract were sufficient to accept the results (Table 2). Please specify the concentration of Miang extracts that were used.

**Response 3:** Thank you for the comment. The concentrations have been indicated as recommended, “YTL: Young tea leaves (109.5 mg/mL), MTL: Mature tea leaves (100.5 mg/mL), NFP: non-filamentous fungi growth based process fermented Miang (74.5 mg/mL), FFP: filamentous fungi-growth based process fermented Miang (91.5 mg/mL). Different alphabets represent significant differences (*p*<0.05, Independent samples T-test” (**LNs 313-317**).

**Comment 4:** Please modify the titles of all figures to improve comprehension.

**Response 4:** Reviewer 1 made the same recommendation as well. The figure titles have been modified appropriately.

**Comment 5:** In the conclusion section, the authors should summarize the optimal concentration of each Miang extract that can be employed to inhibit bacterial growth based on the MIC:MBC ratio of Miang extracts.

**Response 5:** Thank you for the suggestion. We chose not to summarize the optimal concentration of each Miang extracts in the conclusion section because a future study will have to optimize the extracts in an oral formulation or mouthwash then test against oral pathogens. This study merely serves as a prelude and confirms the feasibility of subsequent studies.

**Reviewer 3**

**General Response:** Thank you very much for the comment. Your questions will be addressed appropriately.

**Comment 1:** Line 126 to 136 talks about ‘Determination of antibacterial activities of neutralized (pH 7) and non-neutral (pH 4.0 – 4.6) Miang extracts’. The description, however, misses out on how these extracts were obtained before using them for the microbial tests. The ‘Preparation of Miang extracts’ is also silent on how the neutral and non-neutral extracts were obtained. Authors should kindly take a look at that.

**Response 1:** Now **LNs 131 – 132**, it has been changed to “Determination of antibacterial activities of neutralized (pH 7) and non-neutralized (pH 4.0 – 4.6) Miang extracts”. The preparation of Miang extracts has been modified (LNs 121-122) to address these concerns and “For the antimicrobial tests, an aliquot of each extract was prepared and the pH was adjusted to neutral using 0.1 M NaOH.” was also added.

**Comment 2:** What was the rationale for testing neutralized and non-neutralized extracts in the study? Could the justification be clearly demonstrated in the write-up?

**Response 2:** Thank you for the comment. The question has been addressed in **LNs 321 – 324**, “It was important to test the antibacterial activities of the extracts at non-neutralized and neutralized pH because it showed the observed activities was not exclusively due to low extract pH. Moreover, the pH of the tannin-free extract was adjusted to 5 which is still within the acceptable criteria for mouthwash (4.0 – 6.5) [28]”.

**Comment 3:** Line 127 – I guess ‘non-neural’ should be ‘non-neutral’.

**Response 3: LNs 131 – 132**, changed to “Determination of antibacterial activities of neutralized (pH 7) and non-neutralized (pH 4.0 – 4.6) Miang extracts”.

**Comment 4:** Kindly take a second look at the format for writing your units. At one point, I see CFU/mL, then at other places, I see CFU mL-1.

**Response 4:** CFU/mL has been applied throughout for uniformity (**LNs 147 and 273**).

**Comment 5:** Line 163-164: authors should kindly the sentence ‘The separation of the chemical compounds was be conducted……..’

**Response 5: LNs 168 – 169**, the sentence has been corrected by deleting ‘be’.

**Comment 6:** From Table 2, the zone of inhibition for tannin-rich and tannin-free extracts against Streptococcus mutans were significantly different for each type of sample. How do you authors then arrive at the observation that ‘tannin-free NFP and FFP extracts had significantly (p> 0.05) comparable zones of inhibition with the tannin-rich extracts’ and refer to figure 4, which I suppose is the primary source of data for Table 2? Based on the observations in Table 2, it would be appreciated if authors could reconsider the point on tannin-free producing similar effects as tannin-rich extracts. That obviously also affect the reasoning that

**Response 6:** Thank you for this comment. It was an oversight and the sentence has been corrected to, “However, tannin-free extracts still maintained potent inhibitions against *S. mutans* DMST 18777 (Fig 4)” in **LNs 319 – 321**.

**Reviewer 4**

**General Response:** Thank you very much for the comment. Your concern will be appropriately addressed.

**Comment 1:** This article explain on the antibacterial activity of Miang extract against selected foodborne pathogens and the finding showed that the extract had the ability to inhibit the growth of pathogens. However, there is no problem statement regarding foodborne pathogens even though at the author mention about foodborne problem at the beginning. Author is suggested to do proof read on their article content. This research give new info on the antibacterial activity of Miang extract.

**Response 1:** Thank you for the comment. The title and introduction of the manuscript clearly stated the focus of this research as we aimed to show that the fermented tea could not only inhibit foodborne pathogens (*Bacillus cereus, E. coli* and *Salmonella enterica*) but the tannin-free extracts could importantly inhibit *S. mutans* and potentially inhibit its biofilms. Then, we suggested that these extracts could potentially find applications as ingredients in mouthwashes. However, the mention of “foodborne pathogens” in the abstract has been erased to avoid ambiguity.

**Comment 2:** Line 58- The statement mention that resilient efforts to combat infectious diseases and pathogenic bacteria (such as Bacillus cereus and Escherichia coli) that cause foodborne illnesses and/or pose serious problems to human health is a current research quest. However, the reference use to support the statement that mention ‘current research’ is on 2017. Pls find latest references.

**Response 2:** The reference [3] on LNs 519-523, has been updated to a latest paper, “Chinemerem Nwobodo D, Ugwu MC, Oliseloke Anie C, Al-Ouqaili MTS, Chinedu Ikem J, Victor Chigozie U, et al. Antibiotic resistance: The challenges and some emerging strategies for tackling a global menace. Journal of Clinical Laboratory Analysis. 2022; 36(9):e24655”.

**Comment 3:** The introduction focus on foodborne pathogens issue. However this research do analysis on Streptococcus mutans. Does this strain is classify as foodborne pathogens? State the justification on the selected pathogens.

**Response 3:** Thank you for the comment. Considering Miang is a fermented food that’s orally consumed, we wanted to show that it had the potential to inhibit bacterial pathogens capable of causing foodborne infections (*Bacillus cereus, E. coli* and *Salmonella enterica*). As the extracts showed excellent inhibition against, *S. mutans*, their potential as ingredients of mouthwash was evaluated. The idea was any pathogen that can cause infestation via the gastrointestinal tract can be potentially targeted by Miang.

**Comment 4:** Line 112- Justification on selection of acetone as extraction solvent. We have several types of solvent that normally used in extraction including methanol, ethanol, water and so on.

**Response 4:** This study built on from a paper we published earlier in 2021 where unpublished data from both studies showed acetone had better phenolic contents and antioxidant activities compared to ethanol, methanol and ethyl acetate. (<https://doi.org/10.3390/antiox10071144>). However, we do think it wise and appropriate to optimize ethanol, methanol or water in future studies where mouthwash formulations containing Miang will be prepared and tested.

**Comment 5:** There is no procedures on the adjustment of extract pH

**Response 5:** Reviewer 3 raised a similar question and it was addressed in Lines 121 – 122 of the Materials and Methods, “For the antimicrobial tests, an aliquot of each extract was prepared and the pH was adjusted to neutral using 0.1 M NaOH”.

**Comment 6:** Line 245 and 251. The figures title are similar. Revise the title by adding the pH use because the both figure display antibacterial activity of extract at different pH.

**Response 6:** Thank you for the comment, the figure titles were modified based on recommendations from reviewer 1. In addition, a modification was also added to Table 1 to clarify this better.

**Reviewer 5**

**General Response:** Thank you very much for recommending publishing of this manuscript.
