# Supplementary material for: Antibacterial activities of Miang extracts against selected pathogens and the potential of the tannin-free extracts in the growth inhibition of Streptococcus mutans
Source: PLoS One. 2024 May 8;19(5):e0302717. doi: 10.1371/journal.pone.0302717 (PMC11078415; doi:10.1371/journal.pone.0302717)
Supplement: Attachment — Submitted filename: Response to Academic editor_R2.doc [file pone.0302717.s006.doc]

**POINT BY POINT RESPONSE TO THE EDITOR AND REVIEWERS’ COMMENTS ON THE MANUSCRIPT (PONE-D-23-35234).**

Thank you again for giving us the opportunity to revise the manuscript and the willingness to accept the article after this minor revision. We have considered each of the comments given by both the Academic editor and reviewers and have provided revisions/responses to each comment in a point-by-point pattern. Please note that all corrections by the authors are made using Tracked changes and saved as “Revised document with Track changes”. Line numbers (LNs) are also included in the point by point responses to reviewers for efficient tracking of corrections.

**Academic Editor**

**General Response:** We are grateful to the editor for the kind comments and suggestions meant to improve the manuscript.

**Comment 1:**  Please include a rebuttal letter that responds to each point raised by the academic editor and reviewer(s). You should upload this letter as a separate file labelled 'Response to Reviewers'.

**Response 1:** A rebuttal letter labelled 'Response to Reviewers' has been included per directive of the editor

**Comment 2:**  Please include a marked-up copy of your manuscript that highlights changes made to the original version. You should upload this as a separate file labelled 'Revised Manuscript with Track Changes'.

**Response 2: “**Track changes” in MS word has been used to highlight the revisions and the file has been saved as 'Revised Manuscript with Track Changes'.

**Comment 3:**  Please an unmarked version of your revised paper without tracked changes. You should upload this as a separate file labelled 'Manuscript'.

**Response 3:** A clean copy of the Manuscript devoid of highlights and tracked changes has been labelled and saved as 'Manuscript'.
